# Development of a universal real-time RT-PCR assay for detection of pan-SARS-coronaviruses with an RNA-based internal control

**DOI:** 10.3389/fmicb.2023.1181097

**Published:** 2023-05-18

**Authors:** Beibei Yu, Changping Xu, Shiwang Huang, Jun Ni, Jiancang Zhou, Yuting Zhang, Maomao Wu, Jun Zhang, Lei Fang

**Affiliations:** ^1^Department of Clinical Laboratory, Sir Run Run Shaw Hospital, Zhejiang University College of Medicine, Hangzhou, China; ^2^Key Laboratory of Precision Medicine in Diagnosis and Monitoring Research of Zhejiang Province, Hangzhou, China; ^3^Zhejiang Provincial Center for Disease Control and Prevention, Hangzhou, China; ^4^Shangcheng District Center for Disease Control and Prevention, Hangzhou, China; ^5^Department of Critical Care Medicine, Sir Run Run Shaw Hospital, Zhejiang University College of Medicine, Hangzhou, China; ^6^Key Laboratory of Microbial Technology and Bioinformatics of Zhejiang Province, Hangzhou, China; ^7^Wenzhou Center for Disease Control and Prevention, Wenzhou, China

**Keywords:** molecular diagnostics, SARS-CoV-2, animal coronaviruses, SARS, COVID-19, real-time RT-PCR

## Abstract

The current pandemic caused by severe acute respiratory syndrome coronavirus 2 (SARS-CoV-2) exemplifies the critical need for rapid diagnostic assays to prompt intensified virological monitoring both in human and wild animal populations. To date, there are no clinical validated assays for pan-SARS-coronavirus (pan-SARS-CoV) detection. Here, we suggest an innovative primer design strategy for the diagnosis of pan-SARS-CoVs targeting the envelope (*E*) gene using reverse transcription-quantitative polymerase chain reaction (RT-qPCR). Furthermore, we developed a new primer–probe set targeting human β_2_-microglobulin (*B2M*) as an RNA-based internal control for process efficacy. The universal RT-qPCR assay demonstrated no false-positive amplifications with other human coronaviruses or 20 common respiratory viruses, and its limit of detection (LOD) was 159.16 copies/ml at 95% detection probability. In clinical validation, the assay delivered 100% sensitive results in the detection of SARS-CoV-2-positive oropharyngeal samples (*n* = 120), including three variants of concern (Wuhan, Delta, and Omicron). Taken together, this universal RT-qPCR assay provides a highly sensitive, robust, and rapid detection of SARS-CoV-1, SARS-CoV-2, and animal-derived SARS-related CoVs.

## Introduction

Emerging and reemerging coronaviruses pose serious threats to global health. In December 2019, a cluster of patients suffering from unidentified pneumonia was recognized in Wuhan, China (Zhu et al., 2020). A novel coronavirus was later isolated from a patient's bronchoalveolar-lavage fluid and designated as severe acute respiratory syndrome coronavirus 2 (SARS-CoV-2) by the International Committee on Taxonomy of Viruses (ICTV; Gorbalenya et al., 2020; Wu et al., 2020a). The coronavirus disease 2019 (COVID-19) pandemic has spread rapidly and devastated nearly the entire world, marking the third introduction of a highly contagious coronavirus into the human population in the twenty-first century (Drosten et al., 2003; Zaki et al., 2012; Wu et al., 2020b).

The genome of SARS-CoV-2 is ~29–30 kb in length including the 3′- and 5′-untranslated regions, and the genes toward the 3′-end encode key structural proteins of the spike (S), envelope (E), membrane (M), and nucleocapsid (N; Yan et al., 2020). S protein contains major immunogenic epitopes, which directly bind to angiotensin-converting enzyme 2 (ACE2) for host invasion in a similar manner as SARS-CoV (Raj et al., 2013; Kesheh et al., 2022). Since the RNA genome of SARS-CoV-2 is prone to mutate in the replication process, a number of noteworthy mutants have been identified including Alpha (Leung et al., 2020), Beta (Makoni, 2021), Gamma (Faria et al., 2021), Delta (Singh et al., 2021), and Omicron mutants (Cui et al., 2022). Among them, several mutations in the S protein have been reported to confer enhanced infection efficiency and immune evasion ability (Harvey et al., 2021; Liu et al., 2022; Malato et al., 2022). The genetic and antigenic diversity in SARS-CoV-2 inevitably complicates medical countermeasures to constrain the ongoing pandemic.

Moreover, SARS-CoV-2 is speculated to have a zoonotic origin and maintained in a sylvatic cycle of transmission between wild mammals and cave-dwelling bats as assumed for SARS-CoV-1 (2003; Calisher et al., 2006; Zhou et al., 2020). SARS-CoV-2 may transmit to humans directly through an unknown intermediate mammalian host, such as a pangolin (Johansen et al., 2020). The ubiquitous shedding of coronaviruses in bats and wild animals, along with the anthropogenic interference with natural ecosystems, may facilitate the emergence of a hypothetical SARS-CoV-3 or novel SARS-related CoVs to cause large outbreaks on an international scale (Wang and Anderson, 2019; Irving et al., 2021; Lauring and Hodcroft, 2021; Shivaprakash et al., 2021). From this perspective, the development of an accurate and rapid diagnostic assay to curb the spread of SARS-CoV-2 and improve the capacity for emergency management is urgently needed.

Currently, several kinds of COVID-19 diagnostic tests are available in clinical settings with variable diagnostic accuracy, including molecular assays and antigen detection assays. While the virus has been detected using a variety of immunodiagnostic methods such as immunofluorescent, chemiluminescent, lateral flow, and enzyme-linked immunosorbent assays, few have been widely adopted for large-scale testing due to inconsistent accuracy and heterogeneous sensitivity (Fong et al., 2020). Nucleic acid testing by reverse transcription-quantitative polymerase chain reaction (RT-qPCR) remains the gold standard technique for COVID-19 diagnosis (Liu et al., 2020; Sharfstein et al., 2020). Previous virological studies have made modified assays with specific oligonucleotide probes to achieve SARS-CoV-2 discrimination, but these strategies may not suffice to detect SARS-CoV-1 or SARS-related CoVs. In addition, these RT-qPCR assays generally lack or simply employ DNA as intrinsic control, which cannot minimize the RT-qPCR failure caused by the reverse transcription step. Therefore, the objective of this study is to develop a robust pan-SARS-CoV assay with the optimization of an RNA-based internal control. This universal RT-qPCR assay may have greater potential for the routine surveillance of SARS-CoV-2 variants and to predict future emerged/reemerged SARS-related CoVs or SARS-CoV-1 for global preparedness.

## Materials and methods

### Viruses

Respiratory viruses including SARS-CoV-2 (Wuhan, Delta, and Omicron variants), common human coronaviruses (229E, OC43, NL63, and HKU1), human rhinovirus (HRV), measles virus (MV), human bocavirus (HBoV), human metapneumovirus (HMPV), respiratory syncytial viruses (RSV-A and RSV-B), parainfluenza viruses (PIV1-4), influenza A viruses (H_1_N_1_ and H_3_N_2_), influenza B viruses (Victoria and Yamagata subtypes), mumps virus (MUV), and rubella virus (RV) were stored by Zhejiang Provincial Center for Disease Control and Prevention. The SARS-CoV-2 isolate stock [1 × 10^5^ 50% tissue culture infective doses (TCID_50_)/ml] was titrated on Vero E6 cells as previously described (Gao et al., 2020). All viruses were stored at −80°C until use. The collection, transportation, storage, and detection of viruses were strictly followed by the SARS-CoV-2 Laboratory Biosafety Guidelines (Second Edition; National Health Commission, 2020) and the Technical Guide for Laboratory Testing of COVID-19 (Ninth Edition; National Health Commission, 2022) issued by the General Office of National Health Commission. All SARS-CoV-2 culture or nucleic acid extraction work was performed in a biological safety cabinet of the biosafety level-3 laboratory.

### Nucleic acid extraction

Total nucleic acids were extracted from 200 μl of viral cultures using the RNeasy Mini Kit (Qiagen, Hilden, Germany) according to the manufacturer's specifications. The extracts were eluted into 50 μl of DNase- and RNase-free water, followed by storing at −80°C until assayed for RNA quantitation.

### Primer and probe design for pan-SARS-CoVs

Full-length sequences of SARS-CoV-1, SARS-CoV-2, and animal-derived SARS-like CoV were retrieved from the GISAID database (https://gisaid.org/) and GenBank database (https://www.ncbi.nlm.nih.gov/genbank/; Supplementary Table 1). These sequences and SARS-CoV-2 primers and probes from worldwide available panels (Table 1) were included in the alignment and systematically analyzed the impact of their nucleotide mismatches on pan-SARS-CoVs detection using DNAMAN version 6.0 software. After homology comparisons, conserved DNA segments that cover all downloaded sequences were selected as candidates for primer and probe design. Conserved positions were then evaluated for their role in the GC content, amplicon size, melting temperature (*Tm*), homo and heterodimerization of the primers, and presence of GC clamps at the 3′-end to fulfill the primer and probe design criteria. Three primer-probe pairs (pan-SARS-E, pan-SARS-N, and pan-SARS-ORF1ab) targeting the nucleocapsid protein (*N*), envelope protein (*E*), and open reading frame (*ORF*) 1ab genes were selected as candidate gene regions for further characterization (Table 2).

**Table 1:** Summary of publicly available primer and probe sequences used for SARS-CoV-2 RT-qPCR assays.

**Country**	**Target gene**	**Primer and probe name**	**Sequence (5^′^ → 3')**	**References**
China	*ORF-1ab*	CDC-F	CCCTGTGGGTTTTACACTTAA	China_CDC, 2020
		CCDC-P	FAM-CCGTCTGCGGTATGTGGAAAGGTTATGG-BHQ1	
		CCDC-R	ACGATTGTGCATCAGCTGA	
	*N*	CDC-N-F	GGGGAACTTCTCCTGCTAGAAT	China_CDC, 2020
		CDC-N-P	FAM-TTGCTGCTGCTTGACAGATT-TAMRA	
		CDC-N-R	CAGACATTTTGCTCTCAAGCTG	
	*ORF-1b*	HKU-F	TGGGGYTTTACRGGTAACCT	Chu et al., 2020
		HKU-P	TAGTTGTGATGCWATCATGACTAG	
		HKU-R	AACRCGCTTAACAAAGCACTC	
	*N*	HKU-F	TAATCAGACAAGGAACTGATTA	Chu et al., 2020
		HKU-P	GCAAATTGTGCAATTTGCGG	
		HKU-R	CGAAGGTGTGACTTCCATG	
USA	*N*	USA-F-1	GACCCCAAAATCAGCGAAAT	US_CDC, 2020b
		USA-P-1	FAM-ACCCCGCATTACGTTTGGTGGACC-BHQ1	
		USA-R-1	TCTGGTTACTGCCAGTTGAATCTG	
	*N*	USA-F-2	TTACAAACATTGGCCGCAAA	US_CDC, 2020b
		USA-P-2	FAM-ACAATTTGCCCCCAGCGCTTCAG-BHQ1	
		USA-R-2	GCGCGACATTCCGAAGAA	
	*N*	USA-F-3	GGGAGCCTTGAATACACCAAAA	US_CDC, 2020b
		USA-P-3	FAM-AYCACATTGGCACCCGCAATCCTG-BHQ1	
		USA-R-3	TGTAGCACGATTGCAGCATTG	
German	*RdRp*	GER-F	GTGARATGGTCATGTGTGGCGG	Corman et al., 2020
		GER-P1	CAGGTGGAACCTCATCAGGAGATGC	
		GER-P2	CCAGGTGGWACRTCATCMGGTGATGC	
		GER-R	CARATGTTAAASACACTATTAGCATA	
	*E*	GER-F	ACAGGTACGTTAATAGTTAATAGCGT	Corman et al., 2020
		GER-P	FAM-ACACTAGCCATCCTTACTGCGCTTCG-BBQ	
		GER-R	ATATTGCAGCAGTACGCACACA	
	*N*	GER-F	CACATTGGCACCCGCAATC	Corman et al., 2020
		GER-P	FAM-ACTTCCTCAAGGAACAACATTGCCA-BBQ	
		GER-R	GAGGAACGAGAAGAGGCTTG	
Paris	*RdRp*	Paris-IP2-F	ATGAGCTTAGTCCTGTTG	Institute Pasteur P, 2020
		Paris-IP2-R	CTCCCTTTGTTGTGTTGT	
		Paris-IP2-P	Hex-AGATGTCTTGTGCTGCCGGTA-BHQ-1	
	*RdRp*	Pairs-IP4-F	GGTAACTGGTATGATTTCG	Institute Pasteur P, 2020
		Pairs-IP4-R	CTGGTCAAGGTTAATATAGG	
		Pairs-IP4-P	FAM-TCATACAAACCACGCCAGG-BHQ-1	
Japan	*N*	Jap-F	AAATTTTGGGGACCAGGAAC	Nao et al., 2020
		Jap-R	TGGCAGCTGTGTAGGTCAAC	
		Jap-P	FAM-ATGTCGCGCATTGGCATGGA-BHQ1	
Thailand	*N*	Thailand-F	CGTTTGGTGGACCCTCAGAT	Li et al., 2020
		Thailand-R	CCCCACTGCGTTCTCCATT	
		Thailand-P	FAM-CAACTGGCAGTAACCA-BQH1	

**Table 2:** Primer and probe sequences designed for real-time RT-PCR detection of pan-SARS-CoVs.

**Target gene**	**Primer and probe name**	**Length (bp)**	**Sequence (5^′^ → 3^′^)**
*ORF-1ab*	Pan-SARS-ORF1ab-F	24	GGAAAGGTTATGGCTGTAGTTGTG
	Pan-SARS-ORF1ab-R	18	CCGCACGGTGTAAGACGG
	Pan-SARS-ORF1ab-P	24	FAM-AACGGGTTTGCGGTGTAAGTGCAG-BHQ1
*E*	Pan-SARS-E-F	19	ACACTAGCCATCCTTACTG
	Pan-SARS-E-R	20	CACGTTAACAATATTGCAGC
	Pan-SARS-E-P	21	FAM-CGCTTCGATTGTGTGCGTACT-BHQ1
*N*	Pan-SARS-N-F	19	ACATTGGCACCCGCAATCC
	Pan-SARS-N-R	20	GCTTGACTGCCGCCTCTGCT
	Pan-SARS-N-P	25	FAM-CGTGCTACAACTTCCTCAAGGAACA-BHQ1

### Primer and probe design for internal control

**Figure 1:**
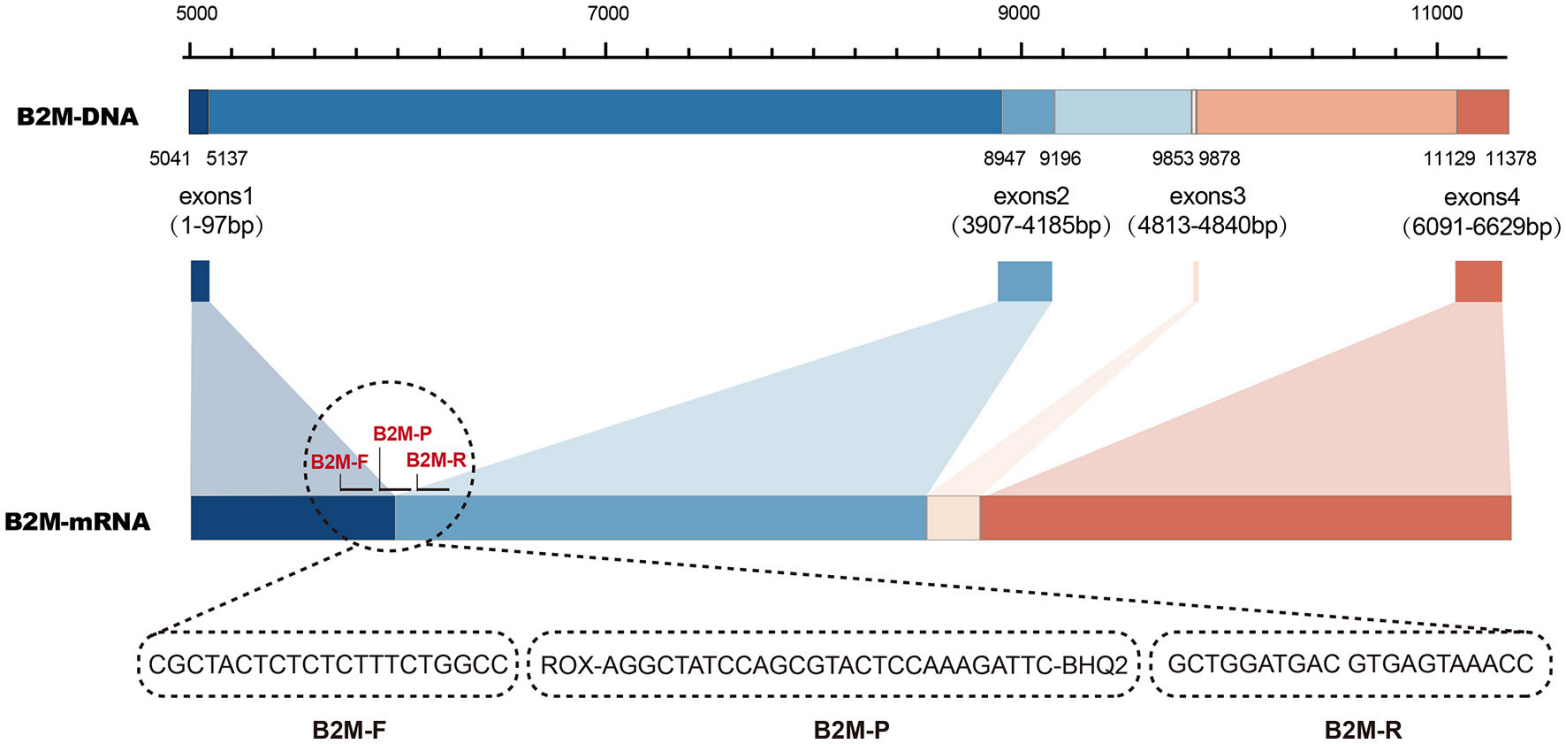
Principle of primer and probe design targeting human beta-2-microglobulin (*B2M*) gene as an intrinsic internal control.

The human housekeeping gene, β_2_-microglobulin (*B2M*), was applied as an endogenous internal control. Its RNA-based primers and probe were designed to span introns to differentiate or eliminate the amplification interference caused by human background DNA. The *B2M* sequence (Accession No: NG_012920.2) and its corresponding mRNA sequence (Accession No: NM_004048.3) were retrieved from the GenBank (https://www.ncbi.nlm.nih.gov/genbank/) database. Sequence analysis was performed by SnapGene and DNAMAN version 6.0 software to identify the starting locus of retained exons. Using *B2M* mRNA as a template, upstream and downstream primers were designed in two distinct exon regions, and the probe was designed to span two exons. The principle of internal control designing and the primer–probe sequences for *B2M* amplification is illustrated in Figure 1.

### Primer and probe selection

All candidate RT-qPCR assays independently tested one COVID-19-confirmed clinical sample using PrimeScript^TM^ RT-PCR (Perfect Real-time) kit (TaKaRa Biotechnology, Japan) by three times to screen the optimum primer-probe set. Each assay was performed in a 25 μl reaction volume containing 12.5 μl of buffer, 0.5 μl of TaKaRa Ex Taq^TM^ HS, 0.5 μl of enzyme mix, 0.48 μM of each primer, 0.24 μM probe, 5 μl of RNase-free water, and 5 μl of RNA template. Amplifications were performed on a 7500 Fast Real-Time PCR system (Applied Biosystems, Waltham, MA, United States) using the following conditions: 10 min at 42°C for reverse transcription, 2 min at 95°C for initial denaturation, followed by 45 cycles of 95°C for 10 s and 55°C for 30 s. The fluorescence signal was acquired at the end of each annealing step. The cycle threshold value (Ct) and fluorescence intensity of designed primer–probe sets were recorded and compared. The candidate assay yielded the lowest Ct-value, and the strongest fluorescence response was selected for further analysis.

### Reaction system optimization

To ensure the sensitivity of RT-qPCR, orthogonal experiments were performed to obtain the optimal concentration of selected primers and probes. The RT-qPCR tests were performed using PrimeScript^TM^ RT-PCR (Perfect Real-time) kit (TaKaRa Biotechnology, Japan), and the amplification cycle was the same as described above.

### Assay specificity and sensitivity analysis

The nucleic acid extracts of SARS-CoV-2 strains and other common respiratory viruses were used as templates to assess the specificity of the pan-SARS-CoV RT-qPCR assay. The nucleic acid extraction of SARS-CoV-2 cell culture isolates was then used to generate standard curves with 10-fold serial dilutions, and each dilution was tested by RT-qPCR assay using both pan-SARS-E and GER-E primer–probe sets for sensitivity comparison. The analytical sensitivity was first determined by the amplification of serial 2-fold dilution of quantified RNA transcripts in 20 replicates/dilution. A commercial quality control product of SARS-CoV-2 with known RNA concentration (Fantasia Biopharma Co. Ltd., China) was selected as a template. PCR water was used as a negative control. Its corresponding limit of detection (LOD) was defined as the concentration (copies/ml) of the lowest dilution at which all replicates could be detected with ≥95% probability.

### Clinical validation

The clinical performance of the universal RT-qPCR assay was parallelly compared with the commercial diagnostic kit (Fosun Diagnostics Co. Ltd., China), which identified the *ORF1ab, N*, and *E* genes specifically for SARS-CoV-2. Primer–probe set of GER-E was also used as a reference for the comparison of clinical performance. A total of 120 remnants of the specimens (oropharyngeal swabs) with low viral load and relatively high C_t_-value (~C_t_-value of 30) were collected from routine monitoring with clinically confirmed COVID-19. The panel included the latest variants of SARS-CoV-2 such as Wuhan-positive (*n* = 50), Omicron-positive (*n* = 60), and Delta-positive (*n* = 10) samples. All patients provided informed consent in accordance with the Declaration of Helsinki.

### Statistical analysis

The result of the RT-qPCR test was considered positive when an exponential fluorescent curve crossed the threshold within 40 cycles. Sensitivity was defined as the proportion of SARS-CoV-2 samples that were detected positive by the RT-qPCR assay; specificity was defined as the proportion of other respiratory viral samples that were tested negative by the RT-qPCR assay. LOD was calculated by a probit regression analysis with a 95% probability endpoint. A 95% confidence interval was provided by the Wilson score method. All statistical analyses were performed using SPSS version 18.0 (Chicago, IL, USA). A *p*-value of 0.05 was considered the minimum level for statistical significance.

## Results

### Primer and probe-template mismatches analysis

**Figure 2:**
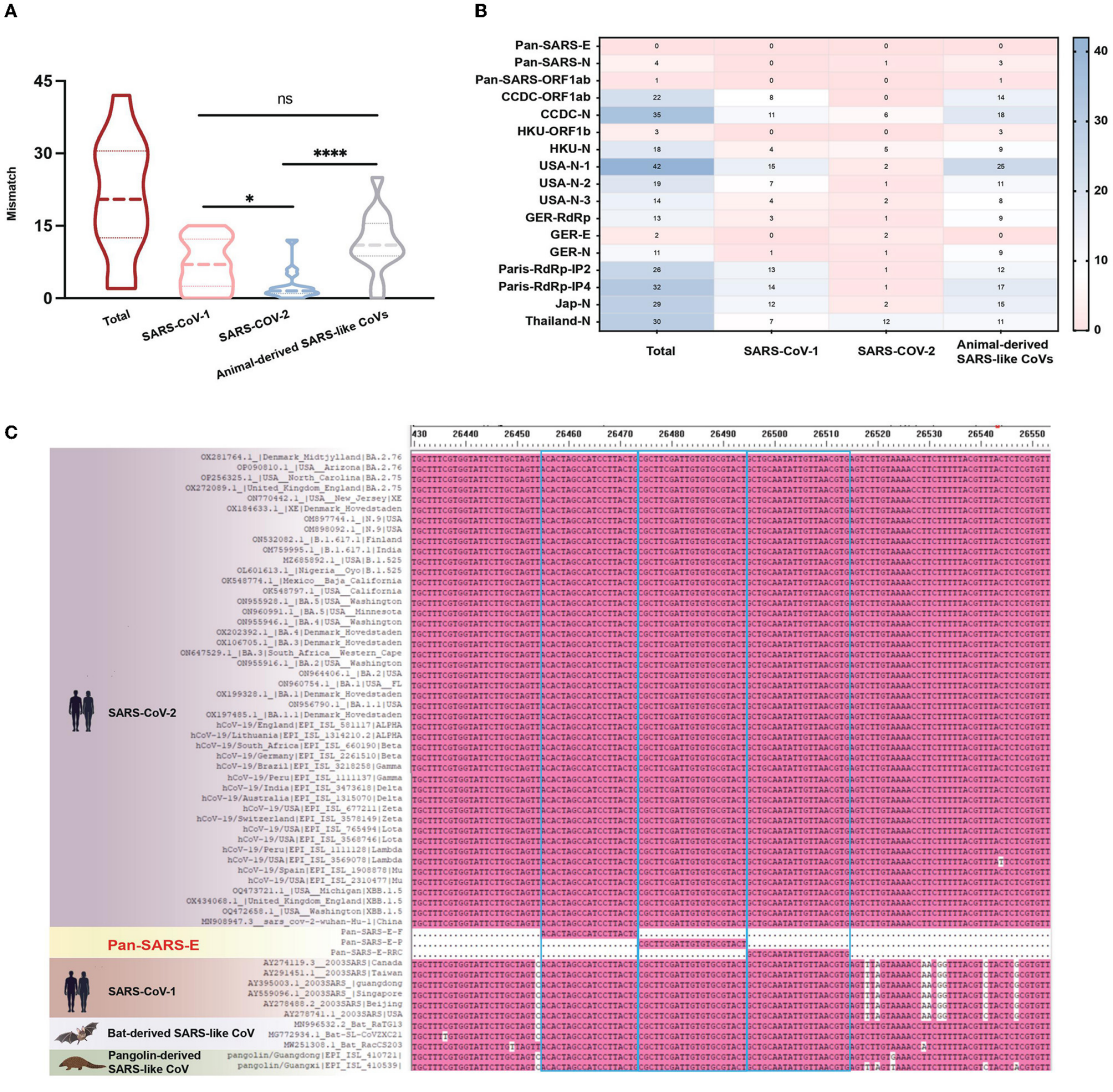
Mismatches in primer and probe binding regions. **(A)** Total and **(B)** individual mismatch patterns of publicly available primer-probe sets against the sequences of SARS-CoV-1, SARS-CoV-2, and animal-derived SARS-like CoVs. **(C)** Mismatches of pan-SARS-E assay in primer and probe binding regions. ^*^P < 0.05, ^****^P < 0.0001, ns, no significance.

As coronaviruses evolve, nucleotide substitutions can occur in primer or probe binding regions and alter the efficiency of RT-qPCR assays. To make a PCR amplification minimally affected by mutations, complete sequences of SARS-CoV-1, SARS-CoV-2 variants, and animal-derived SARS-like CoVs were downloaded from GenBank and GISAID databases, resulting in a final list of 54 sequences. The primer–probe sequences of three candidate assays and several agencies published for SARS-CoV-2 diagnosis were aligned to these 54 genome sequences *in silico* for mismatch examination. Coverage analysis indicated that mismatches were widely distributed in primer and probe binding regions, with a median of 20 nucleotide mismatches for each published primer–probe set (Figure 2A). Publicly available SARS-CoV-2 testing assays displayed significantly more mismatches against the sequences of SARS-CoV-1 or animal-associated SARS-like CoVs than SARS-CoV-2 strains (Figure 2B, Supplementary Figure 1). Conversely, the candidate assay of pan-SARS-E had 100% agreement with the 54 sequences, confirming the good matching of newly designed primers to selected sequences (Figure 2C). Another two candidate assays, pan-SARS-N and pan-SARS-ORF1ab, only showed four and one nucleotide mismatches in binding domains, respectively (Supplementary Figure 2). Of note, the primer–probe set of GER-E (Corman et al., 2020) was highly conserved showing only two mismatches and was selected for later reaction performance comparison.

### Primer and probe selection for the pan-SARS-CoV RT-qPCR assay

**Figure 3:**
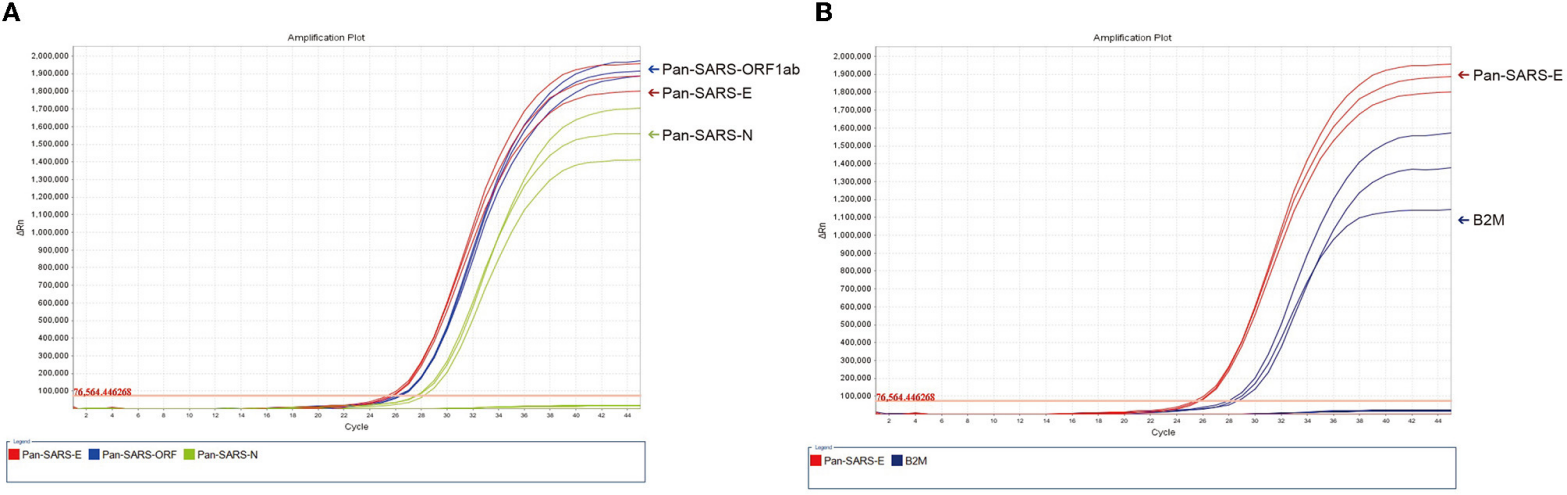
Detection of SARS-CoV-2 RNA by **(A)** three candidate assays (pan-SARS-E, pan-SARS-N, and pan-SARS-ORF1ab) and **(B)** pan-SARS-E with the inclusion of the endogenous internal control, *B2M*.

To validate the diagnostic abilities, the sensitivity of three candidate assays was evaluated by testing SARS-CoV-2 RNA. The mean Ct values were calculated to be 25.72 for pan-SARS-E, 27.92 for pan-SARS-N, and 26.41 for pan-SARS-ORF1ab. All assays were highly sensitive, with the strongest fluorescence intensity achieved by the pan-SARS-E assay (Figure 3A). In the presence of *B2M* internal control, the fluorescence signal of pan-SARS-E remained high for the detection of COVID-19-confirmed human clinical samples and did not generate an interference signal (Figure 3B). Therefore, the pan-SARS-E assay was designated for subsequent optimization and evaluation.

### Optimization of primer and probe concentrations

To avoid probe limitation and ensure maximum sensitivity, the optimal primer–probe conditions for detecting pan-SARS-CoVs were assessed using an orthogonal experimental design. A primer–probe optimization matrix was prepared in which the concentrations of the primers and probes were independently varied and combinatorically tested (Supplementary Table 2). Results showed that the amplification reaction had the lowest Ct-values when the primer-to-probe concentration ratio was 0.4 μM:0.4 μM:0.3 μM (forward:reverse:probe). Taking into account the PCR mixture used in commercial testing kits, the newly designed reaction system was determined to contain 12.5 μl of buffer, 0.5 μl of TaKaRa Ex Taq^TM^ HS, 0.5 μl of RT-qPCR enzyme mixture, 1 μl of 10 μM *E* gene forward primer, 1 μl of 10 μM *E* gene reverse primer, 0.75 μl of 10 μM *E* gene probe, 0.5 μl of 10 μM *B2M* gene forward primer, 0.5 μl of 10 μM *B2M* gene reverse primer, 0.375 μl of *B2M* gene probe, and 5 μl of RNA in a final volume of 25 μl in PCR water.

### Analytical specificity and sensitivity of the universal RT-qPCR assay

The specificity of newly designed primers and probes was first evaluated *in silico* to verify the absence of significant sequence homologies with other respiratory pathogens or human genomes. Then, 20 respiratory viruses that included influenza A (H_1_N_1_ and H_3_N_2_), influenza B (Victoria and Yamagata subtypes), different types of PIVs, RSV-A and RSV-B, HRV, MV, HBoV, HMPV, MUV, RV, and other common human coronaviruses, such as 229E, OC43, NL63, and HKU1, were tested by the pan-SARS-E assay following the same RT-qPCR conditions. All tested results were found to be highly specific for our intended targets without the detection of non-target viruses.

**Figure 4:**
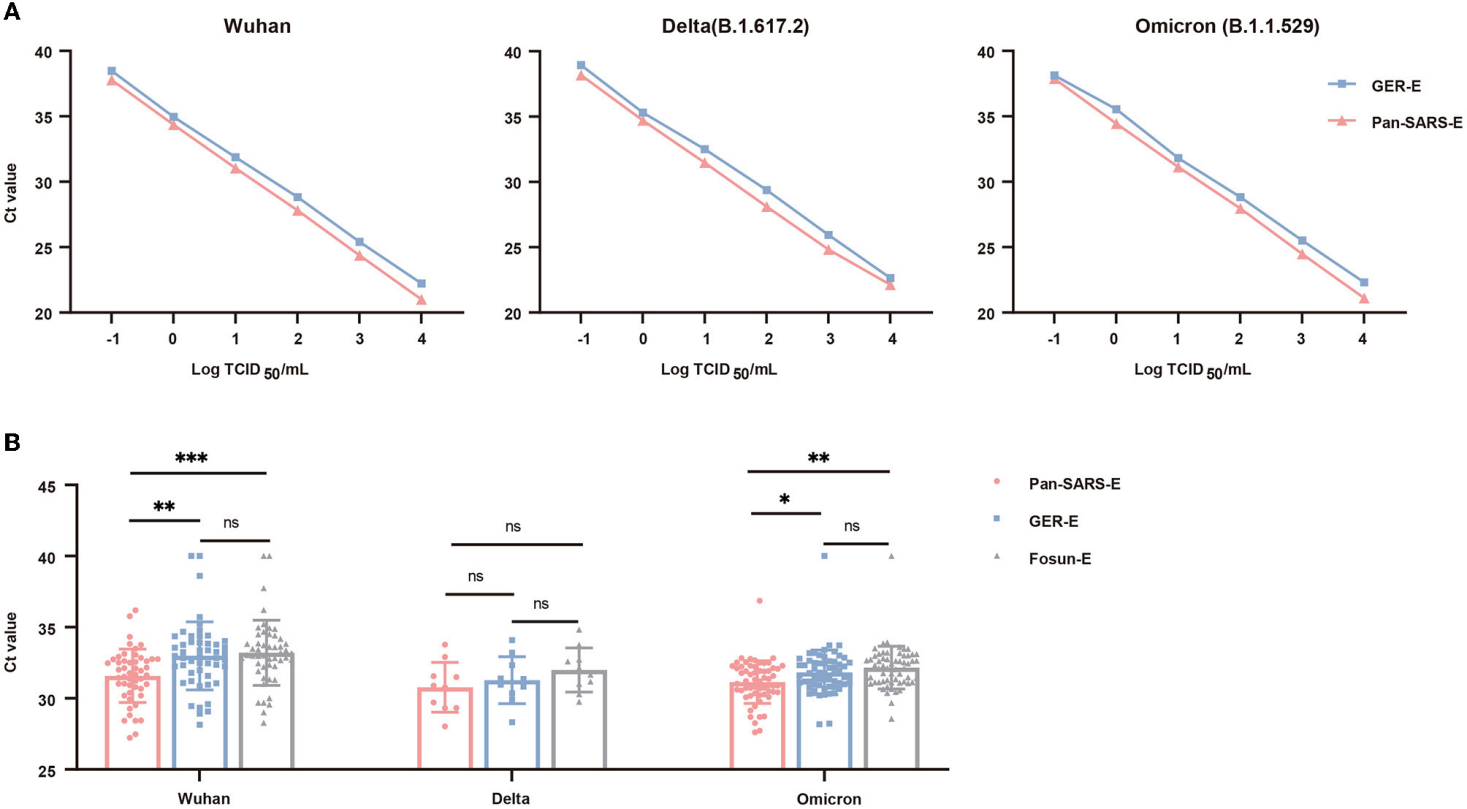
Performance validation of the universal RT-qPCR (pan-SARS-E) assay and approved primer-probe sets for SARS-CoV-2 detection. **(A)** Comparative PCR efficiency of pan-SARS-E and GRE-E assays for testing 10-fold serial dilutions of quantified SARS-CoV-2 RNA. **(B)** Ct-value comparison among pan-SARS-E, GRE-E, and Fosun-E assays for the testing of COVID-19-confirmed clinical samples. Ct-values of the clinical samples with no fluorescent signals were considered 40. *P < 0.05, **P < 0.01, ***P < 0.001, ns, no significance.

To investigate the sensitivity of the optimized RT-qPCR system, we made a direct comparison between the pan-SARS-E assay and GER-E assay, whose primer–probe set exhibited broadly specific features in mismatch analysis. Empirical sensitivity was first evaluated by using a 10-fold serial dilution of quantified SARS-CoV-2 RNAs (ranging from 10^4^ to 10^−1^ TCID_50_/ml). Both assays revealed a strong correlation between RNA concentrations and Ct values (Figure 4A). However, in the same concentration, the pan-SARS-E assay had stronger fluorescence intensity and low C_t_ value than the GER-E assay, indicating that our newly designed RT-qPCR assay was superior to German-published primer–probe set for pan-SARS-CoV detection. We also tested a 2-fold serial dilution of quantified SARS-CoV-2 RNA in 20 replicates/dilution. All 20 tests using quantified RNA at 500 and 250 copies/ml yielded positive results, while 15 and 9 out of 20 tests at 125 copies/ml and 62.5 copies/ml were positive, respectively (Supplementary Table 3). None of the samples using quantified RNA could be detected at 31.25 copies/ml. The LOD of the pan-SARS-E assay was calculated as 159.16 copies/ml of quantified RNA with a 95% probability.

### Clinical evaluation

To further evaluate the diagnostic performance of this novel assay, 120 oropharyngeal swab samples including SARS-CoV-2 Wuhan (*n* = 50), Delta (*n* = 10), and Omicron (*n* = 60) variants were obtained from patients with laboratory-confirmed COVID-19 and were tested using by our novel RT-qPCR assay. The sensitivity of the newly established assay (pan-SARS-E) was assessed and compared to two published primer–probe sets (GER-E and Fosun-E) which also identified the *E* gene. The pan-SARS-E assay exhibited a more significant diagnostic capacity than the other two primer–probe sets and was able to detect 100% of the Wuhan, Delta, and Omicron samples (Figure 4B). All Delta-positive samples had detectable signals for the SARS-CoV-2 *E* gene regardless of assay types, whereas two Wuhan-positive and one Omicron-positive samples failed to be amplified by neither GER-E nor Fosun-E assay (Table 3). Meanwhile, the *B2M* internal control was detected in all clinical samples suggesting a good diagnostic performance.

**Table 3:** Comparison of universal RT-qPCR and commercially available RT-qPCR assays for detection of COVID-19 confirmed clinical specimens.

**SARS-CoV-2 variants**	**Number of samples**	**Sensitivity (%)**		
		**Pan-SARS-E**	**GER-E**	**Fosun-E** ^a^
Wuhan	50	100	96	96
95% Cl^b^		91.11–100	85.14–99.30	85.14–99.30
Delta	10	100	100	100
95% Cl		46.29–100	46.29–100	46.29–100
Omicron	60	100	98.33	98.33
95% Cl		92.50–100	89.86–99.91	89.86–99.91

^a^Commercial COVID-19 detection kit produced by Fosun Diagnostics contains specific primer-probe sets that identify the E, N, ORF1ab genes as domain targets, respectively. Only the results targeting the E gene were used as reference for clinical performance comparison.

^b^95% CI, 95% confidence interval.

## Discussion

The COVID-19 pandemic offers the most recent tragedy when a pathogenic SARS-like sarbecovirus emerges in the human population. Currently, RT-qPCR remains the gold standard for coronavirus detection. Many published primer–probe sets based on real-time detection techniques were provided for SARS-CoV-2 diagnosis (China_CDC, 2020; Corman et al., 2020; Lieberman et al., 2020; US_CDC, 2020a). The outcomes of these SARS-CoV-2 RT-qPCR assays are comparable in sensitivities and can be detected at 500 viral RNA copies per reaction (Vogels et al., 2020). Despite the effectiveness of currently used SARS-CoV-2 diagnostic assays, lessons learned from the COVID-19 pandemic unveil a pressing need to expand the diagnostic landscape for risk monitoring. Since this pandemic was heralded by the 2003 SARS epidemic which promoted the discovery of animal-derived SARS-like CoVs, reliable and inexpensive diagnostic assays which enable both monitoring the circulating SARS-CoV-2 variants and helping against future pandemics are increasingly required for the international community.

**Figure 5:**
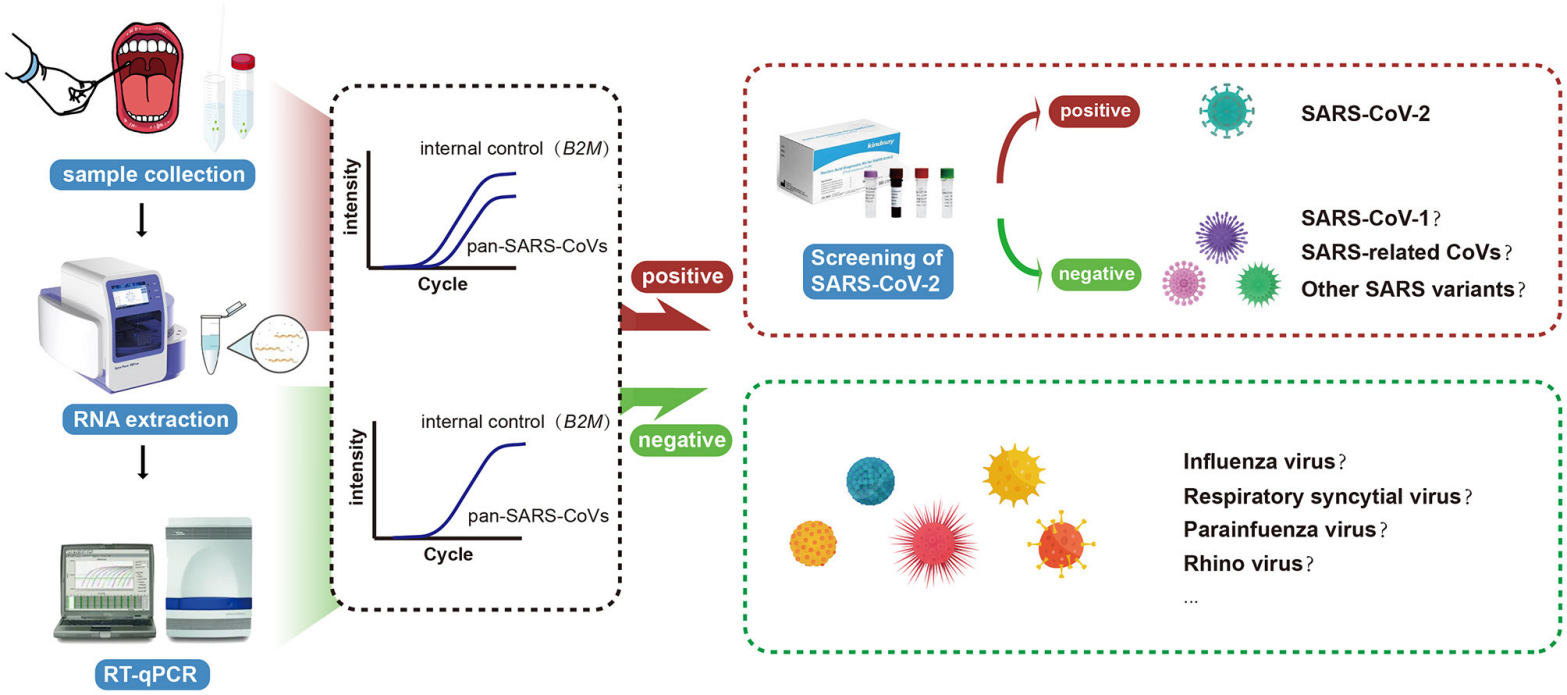
Schematic illustration of the universal RT-qPCR assay for the accurate and rapid detection of pan-SARS-CoVs.

To the best of our knowledge, this is the first RT-qPCR-based assay that was intentionally established for the simultaneous detection of SARS-CoV-1, SARS-CoV-2, and other SARS-like sarbecoviruses to improve the early identification of future emerging novel coronaviruses from this high-risk subgenus or reemerging SARS-CoV-1. The clinical laboratory can adopt a two-tier approach by first attempting to confirm infections using the new pan-SARS-E assay, and subsequently using species-specific assays on clinically suspicious cases to rule out SARS-CoV-2 infections. The diagnostic workflow of the novel RT-qPCR assay is summarized in Figure 5, including the steps and decisions required to perform pan-SARS-CoV detection.

The success of this novel RT-qPCR assay depends first and foremost on the inherent design of pan-SARS-CoV primers. Correct selection and stable hybridization are prerequisites for a primer extension by DNA polymerase. Mismatches affect the stability of primer-template duplex and may alter PCR performance, particularly arising mismatches located in the 3′-end of the primer have a profound detrimental effect on sequence annealing and amplification (Bru et al., 2008; Klungthong et al., 2010; Stadhouders et al., 2010). For example, the reverse primer of RdRp-SARSr (Charité) primer–probe set has one mismatch toward circulating SARS-CoV-2, leading to lower sensitivity and reproducibility than other commonly used SARS-CoV-2 RT-qPCR assays (Corman et al., 2020). We first *in silico* evaluated published primers and probes for SARS-CoV-2 testing against various pan-SARS-CoV sequences. The numerous mismatches among aligning primer and probe sequences with the genomes of SARS-CoV-1 and animal-associated SARS-like CoVs demonstrated that existing detection methods were unsatisfactory to achieve pan-SARS-CoV discrimination. The primary exception to this was the primer–probe set developed by German with the *E* gene target (GER-E), which only had two mismatches and was accordingly chosen for subsequent assessments. To increase discrimination power and amplify more pan-SARS-CoV subtypes, we attempted to deduce broadly specific targets that cover alignments as many as possible. Based on conserved regions, three distinct assays (pan-SARS-E, pan-SARS-N, and pan-SARS-ORF1ab) were initially established.

Then, we performed a parallel assessment of these candidate RT-qPCR assays to detect SARS-CoV-2. The pan-SARS-E assay revealed the strongest signal response probably due to its degenerate nucleotides exactly matching all reference sequences in primer–probe binding regions. The pan-SARS-E assay with the *Sarbecovirus*-specific *E* gene target was, in turn, established by optimizing the reaction system and validated in accordance with the analytical procedures. The optimized pan-SARS-E assay can not only sensitively detect transcripts at levels as low as 159.16 copies/ml but also show no cross-reactivity toward a panel of other common respiratory viruses. During clinical validation, all comparable assays demonstrated high sensitivity above 90%, a “designable sensitivity” threshold set by the World Health Organization (2020); however, our pan-SARS-E assay exhibited superior plasticity to the published primer–probe sets (GRE-E and Fosun-E) with respect to the detection of SARS-CoV-2 variants. Moreover, our results supported that the novel RT-qPCR assay had a great clinical performance compared with prior published diagnostic assays with the *E* gene target to detect SARS-CoV-2 variants (El Wahed et al., 2021; Cherkaoui et al., 2022).

This universal RT-qPCR assay also incorporated a *B2M* internal control, a stable housekeeping gene commonly used for quantitative gene expression analyses (Stamova et al., 2009; Stephens et al., 2011). Internal controls are essential in diagnostic assays to monitor extraction efficiencies and potential PCR inhibitions. While the clinical identification of SARS-CoV-2 using the RT-qPCR method is fairly routine, many laboratories remain faced with strong or mild inhibitors that could impede the amplification of the target (Tali et al., 2021). The specimen type, viral load, transport media, and RNA extraction method are all accounting factors to affect the extent of PCR inhibition in routine examinations which cannot be predicted (Wong and Medrano, 2005; Luebke et al., 2020; Ambrosi et al., 2021; Beltran-Pavez et al., 2021). To ensure PCR accuracy, the *B2M* internal control was added in a half-load concentration of the designed primer-probe set targeting the *E* gene to avoid amplification interference. Another novelty of the current approach was the application of RNA-based internal control to circumvent the problems associated with potential errors and differences generated during cDNA synthesis reactions (Huggett et al., 2005).

Our study inevitably had limitations to consider. The number of oropharyngeal samples for each variant was unequal due to the different circulation features of each SARS-CoV-2 lineage and limited access to obtain clinical samples during the pandemic. Ideally, future studies would be prospective, including SARS-CoV-1-positive samples and SARS-related CoV-positive samples stemming from bats or pangolin to theoretically ensure diagnostic sensitivity. Additional comparisons among multiple types of COVID-confirmed specimens may also be useful for clinical performance validation.

Given the unpredictability of the reemergence/emergence of SARS and other novel coronaviruses from animals or laboratories, this study served as a compelling example with the enhanced testing capacity to improve prognosis. The universal RT-qPCR assay for pan-SARS-CoV diagnosis demonstrated high sensitivity and specificity for detecting 159.16 RNA copies/ml with no observed false-positive reactivity, and its included human *B2M* primer–probe allows for quality control of the whole procedure. This advanced diagnostic technique will ensure continued support for public health, clinical management, and infection prevention and control for SARS-CoV-2 monitoring, as well as provide advice for future pandemic preparedness.

## Data availability statement

The original contributions presented in the study are included in the article/Supplementary material, further inquiries can be directed to the corresponding authors.

## Ethics statement

The studies using human clinical samples were reviewed and approved by the Ethics Committee of the Zhejiang Provincial Center for Disease Control and Prevention. Collection, transportation, storage, and detection of oropharyngeal swabs were strictly followed by the SARS-CoV-2 Laboratory Biosafety Guidelines. All patients provided their written informed consent to participate in this study.

## Author contributions

CX and LF: conceptualization and writing—review and editing. BY, JZ, and YZ: methodology. BY, LF, and JZ: software and writing—original draft. BY, SH, and MW: resources. JN, BY, CX, and LF: data analysis. LF and JZ: project administration. LF, CX, and JZ: funding acquisition. All authors contributed to the manuscript and approved the submitted version.
