# Supplementary material for: Development of a universal real-time RT-PCR assay for detection of pan-SARS-coronaviruses with an RNA-based internal control
Source: Front Microbiol. 2023 May 18;14:1181097. doi: 10.3389/fmicb.2023.1181097 (PMC10232947; doi:10.3389/fmicb.2023.1181097)
Supplement: Supplementary file 1 [file Data_Sheet_1.PDF]

Supplementary Table 1 Summary of SARS-CoV-1, SARS-CoV-2 and animal-derived SARS-like CoV sequences used for comparison

Strain	Accession Number
SARS-CoV-1	AY274119.3, AY291451.1, AY395003.1, AY559096.1, AY278488.2 and AY278741.1,
SARS-CoV-2	Wuhan-Hu-1 (MN908947.3), Eta/B.1.525 (OL601613.1 and MZ685892.1), Kappa/B.1.617.1 (ON532082.1 and OM759995.1), Epsilon/ B.1.427 or B.1.429 (OK548774.1 and OK548797.1), N.9 (OM897744.1 and OM898092.1), BA.1 (ON960754.1 and OX199328.1), BA.1.1 (ON956790.1 and OX197485.1), BA.2 (ON955916.1 and ON964406.1), BA.3 (OX106705.1 and ON647529.1), XE (ON770442.1 and OX184633.1), BA.4 (ON955946.1 and OX202392.1), BA.5 (ON955928.1 and ON960991.1), BA.2.75 (OP256325.1 and OX272089.1), BA.2.76 (OP090810.1 and OX281764.1), Alpha/B.1.1.7 (EPI_ISL_581117 and EPI_ISL_1314210.2), Beta/B.1.351 (EPI_ISL_660190 and EPI_ISL_2261510), Delta/B.1.617.2 (EPI_ISL_3473618 and EPI_ISL_1315070), Gamma/P.1 (EPI_ISL_3218258 and EPI_ISL_1111137), Lamda/C.37 (EPI_ISL_1111128 and EPI_ISL_3569078), Lota/B.1.526 (EPI_ISL_765494 and EPI_ISL_3568746), Mu/B.1.621 (EPI_ISL_1908878 and EPI_ISL_2310477), Zeta/P.2 (EPI_ISL_677211 and EPI_ISL_3578149) and Omicron/XBB.1.5 (OQ472658.1, OQ473721.1 and OX434068.1)
Pangolin-derived SARS-like CoV	EPI_ISL_410721 and EPI_ISL_410539
Bat-derived SARS-like CoV	MN996532.2, MG772934.1 and MW251308.1

Supplementary Table 2 Concentration gradients of primer and probe orthogonally designed for universal RT-qPCR testing

Probe concentration (μM)	0.1	0.2	0.3	0.4
Primer concentration (μM)	Ct value[1]			
0.2	31.59	30.64	30.66	31.16
0.3	30.64	30.32	30.23	30.51
0.4	31.79	30.03	29.83	30.41
0.5	30.39	29.97	30.31	30.19
0.6	31.20	30.19	30.51	30.18
0.7	31.48	30.43	30.72	29.90
0.8	31.46	30.84	31.03	30.05

[1] Ct value = the cycle number where the fluorescence signal crosses the threshold.

Supplementary Table 3 Limits of detection of the universal RT-qPCR assay for detection of SARS-CoV-2 RNA

SARS-CoV RNA concentration (copies/mL)	No. positive tests/no. reaction replicates (%)	95% CI
500	20/20 (100%)	83.9-100%
250	20/20 (100%)	83.9-100%
125	15/20 (75%)	53.1-88.8%
62.5	9/20 (45%)	25.8-65.8%
31.25	0/20 (0%)	0-16%

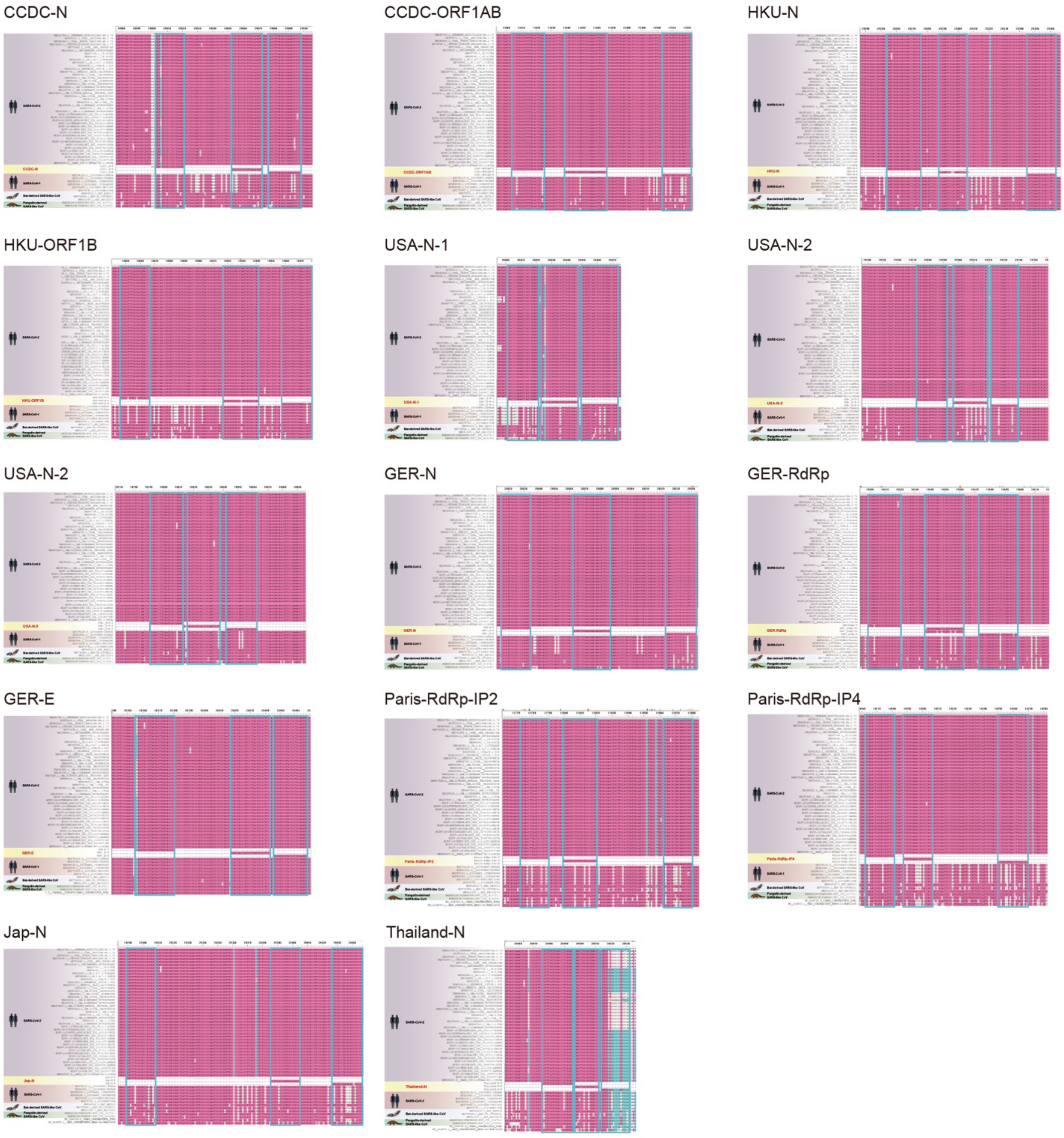

Supplementary Figure 1 The primer and probe-template mismatches among publicly available assays.

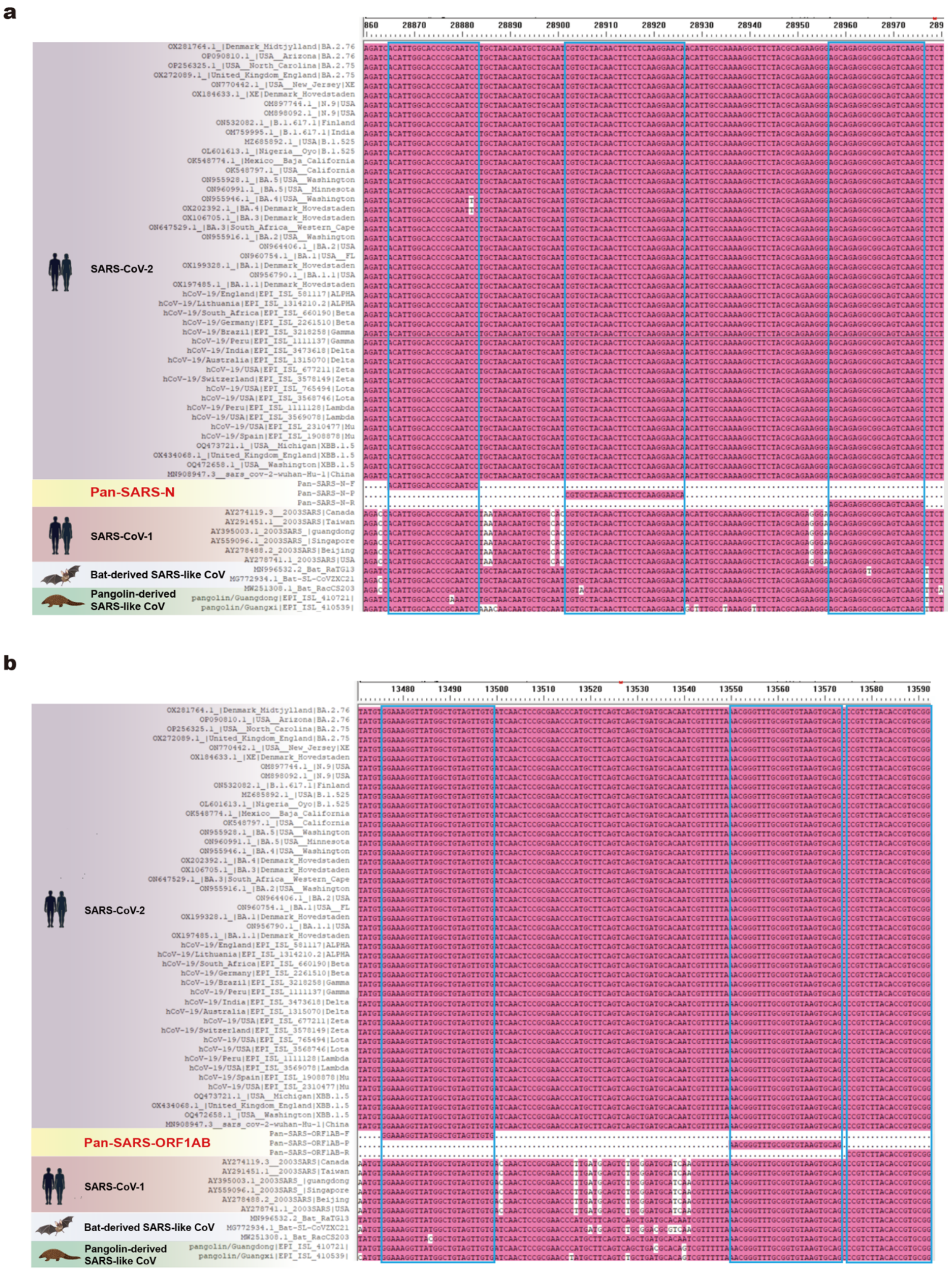

Supplementary Figure 2 Mismatches of candidate assays of **(a)** Pan-SARS-N and **(b)** Pan-SARS-ORF1ab in primer and probe binding regions.
